# Round Shoulders

**Published:** 1892-10

**Authors:** 


### ROUND SHOULDERS.

One of the most frequent deformities against which women must continually fight, especially mothers with young children, is that of round shoulders and a stooping figure. Just observe how many such there are in any gathering. Even the best natural figures will often show this tendency unless some care is taken to prevent it, especially if their work is of such a nature as to keep them sitting at a desk all day, bending over a machine, or doing many kinds of housework ; while thin, narrow-chested women are very likely to stoop before middle age.

Teachers of physical culture say this may be entirely rectified by a very simple and easily performed exercise, that of raising one's self upon the toes leisurely in a perpendicular position several times daily. To do this properly, one must be in a perfectly upright position, with the heels together, and the toes at an angle of 45 degrees, dropping the arms by the side. Inflating and raising the chest to its full capacity is a part of the exercise, a process which the lungs soon begin to show.

To exercise all the muscles of the legs and body one must rise very slowly on the balls of both feet to the greatest possible height and then come again into standing position without swaying the body out of its perpendicular line. This will be the most difficult part of the exercise, but may be accomplished by patience and perseverance. After awhile the same may be tried first on one foot and then on the other.

In order to prevent round shoulders in school children teachers should never ask them to fold their arms in front, but rather to place them behind the back, which occasionally is good practice, giving as it does the fullest expansion to the upper part of the body. For this reason more care is taken now than formerly to see that children sit properly, with the spine kept straight and chest expanded.

